# Expanding theory, methodology and empirical systems at the spatial–social interface

**DOI:** 10.1098/rstb.2022.0534

**Published:** 2024-09-04

**Authors:** Gregory F. Albery, Quinn M. R. Webber, Damien Farine, Simona Picardi, Eric Vander Wal, Kezia R. Manlove

**Affiliations:** ^1^ School of Natural Sciences, Trinity College Dublin, Dublin, Ireland; ^2^ Department of Biology, Georgetown University, Washington, DC, USA; ^3^ Department of Integrative Biology, University of Guelph, Guelph, Ontario, Canada; ^4^ Department of Evolutionary Biology and Environmental Studies, University of Zurich, Zurich, Switzerland; ^5^ Division of Ecology and Evolution, Research School of Biology, The Australian National University, Canberra, Australian Capital Territory, Australia; ^6^ Department of Collective Behavior, Max Planck Institute of Animal Behavior, Radolfzell, Germany; ^7^ Department of Fish and Wildlife Sciences, University of Idaho, Moscow, ID, USA; ^8^ Department of Biology, Memorial University of Newfoundland, St. John’s, Newfoundland, Canada; ^9^ Department of Wildland Resources, Utah State University, Logan, UT, USA

**Keywords:** behavioural ecology, spatial behaviour, social behaviour

## Abstract

All animals exhibit some combination of spatial and social behaviours. A diversity of interactions occurs between such behaviours, producing emergent phenomena at *the spatial–social interface*. Untangling and interrogating these complex, intertwined processes can be vital for identifying the mechanisms, causes and consequences of behavioural variation in animal ecology. Nevertheless, the integrated study of the interactions between spatial and social phenotypes and environments (at the spatial–social interface) is in its relative infancy. In this theme issue, we present a collection of papers chosen to expand the spatial–social interface along several theoretical, methodological and empirical dimensions. They detail new perspectives, methods, study systems and more, as well as offering roadmaps for applied outputs and detailing exciting new directions for the field to move in the future. In this Introduction, we outline the contents of these papers, placing them in the context of what comes before, and we synthesize a number of takeaways and future directions for the spatial–social interface.

This article is part of the theme issue ‘The spatial–social interface: a theoretical and empirical integration’.

## 1. Introduction

All animals are social and all animals interact with their spatial environment. Nearly all biological processes are connected to spatial or social behaviour in some way: for example, learning [1], ageing [2] and disease spread [3,4] all have the potential to affect, or be affected by, some combination of spatial and social factors. Animal space use and sociality are tightly interlinked and reciprocal [5,6], and it is therefore integral to study how and why individuals move, meet and interact in concert to understand the ecological and evolutionary consequences of many types of behavioural variation [7]. Not doing so can have the effect of reducing power, or losing the ability to test exciting questions and possible unforeseen causes and consequences of spatial or social behaviour. To provide a framework for conceptualizing and testing such connections, we recently conceptualized ‘the spatial–social interface’ [5]. The spatial–social interface, and the connections within it, can provide the tools to inform a prodigious range of empirical and theoretical questions and issues of fundamental ecology and evolution [5]. Ultimately, these could help to derive applied solutions to conservation threats, animals’ responses to global change and the spillover of zoonotic disease into humans [3,8]. However, doing so requires substantial theoretical, methodological and empirical advances in the field.

Recent years have shown a sharp increase in empirical studies that quantify the interplay between social and spatial processes. Augmented by a concurrent growth in animal social network analysis [9], movement ecology [10,11] and collective behaviour [12], and availability and sharing of spatial data [13], researchers are identifying links between spatial and social behaviours and environments in a wide range of systems [14–16] and interrogating their consequences more broadly [5,6]. Nevertheless, despite a wide appreciation of their joint importance and a range of identified spatial–social synergies and interactions, a formal conceptual integration of the spatial–social interface is only in its relative infancy [5]. Unified theory, frameworks and tools with which to interrogate the interface are scarce, and its up- and downstream correlates remain unclear. Many studies have been restricted to identifying relatively simple interconnections among spatial and social behaviour themselves. At this stage, providing further evidence for relationships among spatial and social behaviours and their consequences is only one frontier, which by itself is unlikely to be able to make significant further headway. Instead, we must broaden the horizons of spatial–social ecology, capturing the diversity of scales and systems in which these phenomena interact, and using novel tools and approaches to ask precise, theory-inspired questions. Furthermore, conducting such analyses in a range of animal systems, and contrasting these examples to describe contingencies and complexities that arise, is a crucial part of developing and honing the theory underlying spatial–social ecology.

In Webber *et al*. [5], we synthesized the spatial–social interface framework, outlining its four key components: spatial phenotypes, social phenotypes, spatial environments and social environments. As part of this exercise, to extend and challenge the ideas we posited, we organized this theme issue and invited a series of contributions inspired by our rubric. We present four broad article topics. (1) Articles that take a mechanistic approach to assess the cognitive mechanisms underlying the connections between spatial and social behaviour [17,18]. (2) Articles that take an emergent approach to assess how spatial and social phenotypes produce the experienced socio-spatial environment [19–21]. (3) Articles that take a bottom-up approach and assess the population-level consequences of spatial–social behaviours [22–25]. (4) Articles that take a top-down approach to identify external pressures that influence the formation of the spatial–social interface [8,26,27]. These topics effectively split our understanding of the spatial–social interface into mechanisms (topics 1 and 2) and consequences (topics 3 and 4). Specifically, topics 1–2 address finer-scale and within-individual perceptions and mechanisms leading to the generation of the spatial–social interface, while topics 3–4 expand our view beyond the interface at the individual and between-individual level to consider its broader consequences and constraints emerging from variable contexts. Finally, our fifth topic expands the methodological frontier at the spatial–social interface and integrates tools that incorporate social and spatial data [28].

Providing case studies and targeted reviews outlining the state of the art at the spatial–social interface, across a wide range of scales (neurological to macroecological), and across a selection of different study systems, we aim to expand the remit for studies at the spatial–social interface. Furthermore, by spotlighting the broad implications and interlinked nature of the interface with other facets of organismal biology and ecology, we hope to catalyse interest in the topic across a selection of other subfields. As well as cementing the study of spatial–social phenomena as a subfield in itself, these advances will help this subfield to make its maximal impact on both fundamental and applied sciences.

## 2. Section 1: Cognitive drivers and mechanisms of individual social and spatial decisions

Because social and spatial behaviours are controlled by the same nervous system and share a range of conceptual characteristics, their manifestation will likely involve a complex series of interconnected cognitive mechanisms. Nevertheless, the cognitive ecology of the spatial–social interface is poorly understood, and explicit comparisons of the structure and function of social and spatial behaviours are likely to be highly informative in this regard. This theme issue includes two papers to this end, each taking a different perspective on the question: what cognitive mechanisms underlie spatial and social behaviours and their interactions?

### (a) Thompson and Parkinson: Interactions between social and spatial representations in the human brain

In their review, Thompson and Parkinson [17] compare the representations of social and spatial phenomena in the human brain, accentuating that spatial and social comparisons run deep through human language and perception. In discussing morphological similarities between social and spatial concepts in neuroscience, they identify that both follow a similar topological map representation, hinting at deep mechanistic similarities in how the brain processes these types of information. Similarly, they identify interactions between social and spatial concepts, where each component modifies or informs the other—for example, in encoding spatial relationships between locations using socially defined heuristics or groupings. Thompson and Parkinson provide a unique perspective of the spatial–social interface. Specifically, the neural mechanisms associated with social and spatial knowledge are thought to be highly interconnected, yet understanding these tightly entwined neural processes might help to inform the encoding of relational information.

### (b) Lourie *et al*.: Spatial memory obviates following behaviour in an information centre of wild fruit bats

Lourie *et al.* [18] examine the role of social information in governing resource acquisition behaviours in Egyptian fruit bats (*Rousettus aegyptiacus*). They show that social cues play an important role in helping bats to navigate to ephemerally fruiting fig trees (*Ficus sycomorus*). The authors demonstrate that bats are able to use information from colonymates to understand when certain trees whose position they know about, but have no up-to-date information on, are fruiting. This finding merges spatial and social cognitive mechanisms previously observed only in honeybees, and represents an exciting widening of the possible cognitive pathways by which spatial and social cues can be used in combination in wild animals.

## 3. Section 2: The role of social and spatial phenotypes in the experienced environment

An enduring question concerning the spatial–social interface revolves around the interaction between intrinsic and extrinsically generated phenomena. That is, how does an individual’s behavioural phenotype influence their environment and to what degree does this differ for spatial versus social phenotypes that are inherently related to one another? Our issue presents three papers that explore facets of these questions.

### (a) Merkle *et al*.: How spatial–social interface shapes the grazing ecology of Yellowstone bison

Merkle *et al*. [21] examine bison (*Bison bison*) movements in Yellowstone National Park, USA to test how the spatial–social interface influences their grazing ecology. They find the two sides of the interface are able to strongly buffer one another: while high spatial familiarity allowed individual bison to determine their own movements, in its absence, high social familiarity allowed them to use others’ movements to pursue and obtain resources. Such demonstration of mutual phenotypic redundancy could indicate the factors maintaining between-individual variation in behaviour, with cognitive mechanisms representing a possible proximate mechanism underlying this heterogeneity.

### (b) Baker *et al*.: Active crocodiles are less sociable

Baker *et al.* [20] use acoustic arrays to examine the spatial and social behaviours of a population of estuarine crocodiles (*Crocodylus porosus*) over a decade, taking particular care to quantify both within- and between-individual variation and to compare them. They discover notable repeatability of both spatial and social behaviours, as well as linking them together—finding, surprisingly, that more active individuals tended to be less sociable. This contrasts with prior, perhaps more intuitive, findings showing that wider ranging individuals—e.g. deer—tend to make more contacts [15]. Furthermore, by quantifying the degree of variation they examined individuals’ specialization in behaviours, finding that individuals exhibiting stronger tendencies to one end of this syndrome (more sociable or less active) also tended to be more specialized. These findings accentuate that spatial–social behavioural syndromes can be unexpected, and will likely vary substantially when investigated in novel animal taxa and social systems.

### (c) Wood *et al*.: The scale and complexity of Hadza hunter–gatherer spatial behaviour in comparative perspective

Wood *et al*. [19] apply the spatial–social interface in the field of evolutionary anthropology by asking whether Hadza hunter-gatherers in Tanzania share behavioural traits with olive baboons (*Papio anubis*). Specifically, using a comparative approach, the authors test the hypothesis that brain size and socio-spatial behaviours are linked. Hadza foragers rapidly accumulate information about their socio-spatial world, whereas olive baboons tend to have a narrower and more homogenous pool of information about the socio-spatial world. These results hint that greater cognitive capacity might facilitate complexity at the spatial–social interface. Moreover, Wood *et al*. interrogate the evolution of language and storytelling within the context of the spatial–social interface. The authors identify that central place foragers (e.g. Hadza groups) travel widely and disperse during the day, but return in the evening to a central location. Under these conditions, information is shared about the social and spatial features an individual or group encountered throughout the day; an ecological scenario which the authors argue is conducive for the evolution of language. Within the broader context of the spatial–social interface, Wood *et al*. contribute to our understanding of the evolutionary origins of how groups communicate with one another about their social and biophysical environments.

## 4. Section 3: Population-level consequences at the spatial–social interface

Spatial and social behaviours do not manifest solely at the level of the individual, but magnify to have emergent ‘bottom-up’ consequences. In this section, we present four papers that link across these scales to understand the emergent properties of the spatial–social interface.

### (a) Picardi *et al.*: Scale at the spatial–social interface

Picardi *et al.* [22] add a critical perspective to the initial formulation of the spatial–social interface framework by explicitly outlining the role of scale. Scale is a fundamental component of ecology and given the spatial–social interface framework is relatively nascent, the need for further clarity on how we quantify and interpret spatial–social phenomenon across scales is much needed. The authors outline such frameworks in the form of use-availability concepts that are commonly used in spatial ecology, hierarchical models of spatial–social phenotypes and their constraints, and scale of inquiry itself. The astute observation that a given trait could constitute a different component of the interface depending on the implicit scale being considered is vital for the future of the field and framework. Picardi *et al*. provide much for future consideration regarding scale as empiricists continue to adopt the spatial–social framework in their own systems.

### (b) Chimento and Farine: The contribution of movement to social network structure and spreading dynamics under simple and complex transmission

Chimento and Farine [23] examine the interplay between movement and the transmission of information or disease with an aim to estimate how these aspects of the spatial–social interface influence the emergence of social networks. Using simulations of moving and learning individuals based on different spatial and social rules, the authors examine how the combination of movement and learning governs the emergent structure of the social networks and thereby the spread of novel behaviours. Broadly, resource-based and more localized movements generated more skewed, clustered networks, while nomadic and less-localized movements tended to homogenize the network, which then influenced the speed at which the novel behaviours spread across the population. The finding that dynamic networks are required to accurately model these processes is likewise important for our methodological understanding of spatial and social interplays and the progression of future studies. Within the broader context of the spatial–social interface, this study provides insight into how spatial and social phenotypes interact to influence the emergence of a social environment.

### (c) Hendrix *et al.*: Faithful pals and familiar locales: social and spatial site fidelity during reproduction

Examining replicate populations of GPS-tagged caribou (*Rangifer tarandus*), Hendrix *et al*. [24] quantify individual behavioural tactics in terms of both spatial and social fidelity during reproduction. Their findings indicate that site fidelity—proximity to familiar sites—and social fidelity—proximity to familiar conspecifics—are correlated strategies at the individual level, suggesting synergy between spatial and social traits in determining calving site locations. The simultaneous occurrence of spatial and social fidelity may mean that one occurs as a result of the other (e.g. spatial patterns emerging as a result of social behaviours) or that selective pressures favour the combination of social and spatial traits. Although this study failed to detect the fitness effects of individual spatial–social traits in terms of calf survival, evidence from similar systems warrants further investigation of the selection for these traits and the underlying evolutionary forces governing them.

### (d) Pandey *et al*.: The influence of social and spatial processes on the epidemiology of environmentally transmitted pathogens in wildlife: implications for management

Pandey *et al.* [25] use a series of agent-based epidemiological simulations to test how spatial and social behaviours influence pathogen transmission dynamics, when considered in concert with the pathogen’s persistence in the environment. By varying their parameters widely, they identify a number of important interactions between social behaviour, spatial mobility and pathogen persistence, finding complex context dependence among these factors. The paper provides essential evidence that it can be important to quantify both spatial and social behaviours when investigating parasites across a range of transmission modes.

## 5. Section 4: Top-down drivers of the spatial–social interface

Spatial and social behaviours—and, by extension, their interface—occur in the context of the broader environment. They are undoubtedly influenced by this environment, but it is unclear exactly how environmental contingencies produce variation in spatial–social behaviours. The studies in this issue focus on a range of top-down drivers, including social and spatial resources [26], predation [27] and anthropogenic change [8].

### (a) Ricci *et al*.: Movement decisions respond to temporally varying social and biophysical resources in a long-lived ungulate, bighorn sheep (*Ovis canadensis*)

Ricci *et al.* [26] use a wild population of bighorn sheep (*Ovis canadensis*) to test hypotheses about how the resource landscape (in terms of both physical and social resources) determines individual movement decisions. Interestingly, they find that shorter durations for both social and physical factors (mate availability and droughts, respectively) were positively associated with long-distance foray movement decisions, indicating that ephemerality drove greater responsiveness. This study underscores the importance of considering resources, and especially dynamic variation in their availability, when interrogating the motivations underlying spatial and social behaviours.

### (b) Prokopenko *et al*.: Friends because of foes: synchronous movement within predator–prey domains

Prokopenko *et al*. [27] test how socio-spatial behaviours of elk (*Cervus elaphus*) and caribou depend on perceived predation risk. Both social (e.g. aggregation) and spatial (e.g. movement) behaviours in prey species evolved to balance the tradeoff between resource acquisition and predator avoidance. Synchrony of movements amongst conspecifics is a social-spatial phenotype that can act as a powerful antipredator response. This study highlights that movement synchrony is modulated by prey foraging domain and predator hunting domain and mode, with prey exhibiting higher synchrony at times when foraging resources are scarce and predation risk from cursorial predators is high. Furthermore, seasonal patterns in the degree of synchrony indicate that prey synchronize their movement in response to a suite of factors that include not only predation pressure but also forage availability and their own reproductive life cycle.

### (c) Gaynor *et al.*: Anthropogenic impacts on the spatial–social interface

Gaynor *et al.* [8] review the complex topic of the spatial–social interface in the context of a changing world. Casting a broad net, they cover a wide range of human effects on animals’ spatial and social behaviour, linking complex chains of events that traverse the spatial–social interface in multiple directions using a framework that integrates anthropogenic disturbance into the spatial–social interface. For example, they discuss how weather anomalies have influenced herring (*Clupea harengus*) by altering the distribution of resources (spatial environment), which fed into their space use (spatial phenotype), thereby altering the age composition of the population (social environment), which influenced social learning of migration knowledge (social phenotype), with possible consequences for migration routes (spatial phenotype) [29]. This intuitively connected but nevertheless complex chain of events and processes helps to shine a light on the utility of the spatial–social framework, helping us to understand wildlife biology in a rapidly changing world. Ultimately, they synthesize their findings to suggest routes to management interventions for conservation and wildlife management.

## 6. Section 5: Methods

Finally, a productive research field is only as good as its tools, and the development and honing of technology and software has been a vital part of the field’s nascence. To this end, Gahm *et al*. [28] aims to advance the methodological boundaries of the spatial–social interface.

### (a) Gahm *et al*.: Disentangling reference models for social and spatial networks

Gahm *et al.* [28] provide a methodological study that compares null models of tracking data, proposing that a ‘wrap-around’ method provides utility in certain situations over and above a more commonly used ‘path shuffling’ randomization approach. Their agent-based simulations demonstrate that the wrap-around method more accurately reproduces the original tracks, allows distinction between spatial and social drivers of behaviour, and particularly produces fewer false positives. Finally, using tracking data from a wild population of vultures, they then demonstrate the use of their method for testing the drivers of social interactions.

## 7. Future directions

Together, these papers make an important contribution to furthering our understanding of spatial and social behaviour, expanding our knowledge in a variety of dimensions. As we continue moving forward as a research community, we have a number of primary takeaways.

### (a) The value of studying both space and sociality

The studies in this issue revealed an encouraging diversity of types of value when studying the two sides of the spatial–social interface. For example, there was *synergy*: Hendrix *et al.* [24] found that spatial and social fidelity were ‘synergistic, not alternative’ in reproducing caribou. There was *redundancy*: Merkle *et al*. [21] found that spatial and social familiarity could buffer and replace one another in grazing buffalo. And finally, there were *tradeoffs*: Baker *et al*. [20] found tradeoffs between spatial and social specialization in wild crocodiles. Put simply, these examples speak to the rich diversity of dynamics occurring at the interface: it is hard to generalize when identifying why to study space and sociality, but without doubt it is very often worth doing. We are aware of a semi-pervasive interpretation that investigating the spatial–social interface is thought of as important for reasons of rigour, some of which has emerged from our work: for example, that space should be ‘controlled for’ when investigating sociality (e.g. [30]). However, we are strongly of the opinion that this is an unintended, offputting framing of the interface: rather, as argued elsewhere (e.g. [15,31,32]), we think that investigating space in social contexts (and *vice versa*) offers exciting opportunities to test new questions, and in new ways.

### (b) Interrogating theory

Theory is foundational to the synthetic and empirical work presented in this issue. Classical ecological theory provides a foundation through which we may develop, or refine, hypotheses about the spatial–social interface (described briefly in [5]). From an empirical perspective, collecting data or designing experiments should be informed by ecological theory [33]. For example, Grainger *et al*. [33] propose four steps to using theory in ecological research: (i) adopting a framework; (ii) testing predictions; (iii) testing assumptions; (iv) using mathematical equations. While these four steps can be used together, they can also be used independently. We view adopting the spatial–social interface framework as step 1, with many articles in this theme issue testing predictions (e.g. [20,24,26]) and assumptions [28] and using mathematical equations [23]. For our field to remain unified going forward, we propose researchers continue to develop the spatial–social interface as a framework to inform theory, e.g. through interrogating the integration of scale [22].

### (c) Cross-disciplinarity

Cross-disciplinary research represents an exciting future of the spatial–social interface: for example, the opportunity to integrate aspects of cognition and neural encoding into studies of social and spatial behaviour (e.g. Section 1 of this issue). It was particularly exciting to see the merging of anthropology into this field by Wood *et al*. [19]. Another exciting opportunity to identify potential mechanisms driving social and spatial behaviour is the integration of physiological or immunological factors underlying social and spatial behaviour (e.g. physiology-sociality: [34]). Within the context of evolutionary biology, there are a multitude of opportunities to test hypotheses about how social and spatial phenotypes (and genotypes) covary and affect fitness (e.g. [35]). Comparative ecology has a long history within the context of animal space use (e.g. [36]), but is relatively novel for social networks [37]. Future work could leverage existing long-term datasets on both social and spatial behaviour to make broad inferences about the spatial–social interface across systems, populations and species (e.g. [38]).

### (d) Clarifying terminology

Semantics, while potentially dull, can become a pressing issue in contemporary ecology as cross-disciplinary research becomes increasingly common. This is particularly the case when researchers across sub-disciplines use different language to describe similar phenomena. In developing this theme issue, we noticed substantial variation across authorship teams in their use of terms associated with social and spatial ecology. When combining similar terms with varied definitions or interpretations from social, spatial, movement, disease and evolutionary ecology, it could be problematic for our field to move forward. We suggest that authors use consistent terminology, and (for example) that reviewers of future articles refer back to definitions provided in Webber *et al*. [5] and throughout this theme issue to standardise across studies.

### (e) Taxonomic diversity and comparative analyses

Despite being universal to animals, the systems in which the spatial–social interface is investigated still comprise a skewed selection of the animal kingdom. This issue features four studies on ungulates, one on primates, one on bats, one on birds and one on reptiles. The relative rarity of primates and the absence of insects is notable, especially given their enormous importance in the social behaviour literature (e.g. [39,40]). The overrepresentation of ungulates, and the diversity otherwise, is more representative of the spatial behaviour and movement ecology literature, demonstrating that currently the spatial–social interface has generally been adopted in this direction (i.e. from spatial systems to incorporate social aspects). An important priority in the future must be to identify how to incorporate more spatial questions into studies focused primarily on social behaviour, particularly in bird, primate and insect systems. We have previously noticed the dearth of primate data in a spatial–social meta-analysis [38]; relevant to this problem is the similar rarity of comparative work at the spatial–social interface. Comparative analyses are important because they help to identify general (and generalizable) trends [41] and help to account for context dependence. For example, explicitly comparing responses to culling in geese [42] and badgers [43] might be useful for understanding when perturbations like these alter socio-spatial structure (e.g. through dispersal), and how they depend on the aspects of the social system. Several papers in this issue compared species [19,27]; we hope that future studies will continue to incorporate and compare spatial and social processes across species, contexts and scales [22]. Comparative analyses could ask, for example: why does the activity-sociality correlation reported by Baker *et al*. [20] differ from the opposite finding in red deer [15]? Does it emerge purely from biology, or also from aspects of the sampling regime involved? Comparative work is a growing field of social network ecology [37], and merging it with spatial behaviour is bound to be highly productive.

### (f) Methodological advancements and the data frontier

Several areas of potential methodological improvement are highlighted in this issue. The advancing data frontier is still noticeable, in that there are several commonly acknowledged data problems that remain at the spatial–social interface. Most notably, spatial data are commonly used to answer social questions: proximity is used to detect or approximate social interactions, either using direct censuses (e.g. [15]) or remote biologging and telemetry (e.g. [24]). The approach of using spatially explicit data to answer social questions provides a useful workaround to the fact that social interactions can be more difficult to detect than collocations in space, but this approach is still afflicted with problems such as missingness in the data. Regardless, we are optimistic that missing data is not an insurmountable obstacle for spatial–social interface studies. There exist no ‘perfect’ systems in which there are no missing individuals either in the social or the spatial realms, although ATLAS or other reverse GPS methods might offer promising future opportunities. Indeed, there is no perfect system for *any* scientific question, and testing and acknowledging shortcomings like these is ultimately likely to be more fruitful than searching for a Holy Grail of Data. To that end, owing to the lack of comparative work (§7e) it is unclear how sampling biases like these impact the relationships that we detect between spatial and social phenotypes and environments. That is, co-sampling biases are still a complete black box. Perhaps future studies will build further on the rapid growth in spatial data to reduce biases, or build on ongoing efforts to develop pipelines for verifying data collection approaches’ vulnerability to them [44]. There is already some agreement across fields: for example, Chimento and Farine [23] call for dynamic networks, as previously suggested [37], pointing towards universal data objectives. Building off the spatial–social interface, researchers may use information from both spatial and social dimensions to determine which individuals to tag in a population, e.g. through targeted tagging of multiple individuals in multiple social groups to answer questions about within and between-group sociality and spatial partitioning. This approach could improve methodology on both sides of the spatial–social interface.

### (g) Applied outputs

Throughout this issue, we have expanded the empirical and theoretical remit for the spatial–social interface by explicitly conveying the applied relevance of this burgeoning field. For example, several studies [8,23,25] mentioned implications of the spatial–social interface for disease dynamics, which could be used to inform (and ultimately, to reduce) transmission of pathogens among animals in wild and domestic populations, and to prevent their transmission into human populations [3]. Gaynor *et al*. [8] include a substantial focus on conservation and ecosystem function and offer exciting potential routes for our lessons from the interface to become maximally useful. Applied work at the spatial–social interface will offer an exciting blueprint for translating this work for managers, conservationists, epidemiologists, and more.

## 8. Conclusion

Although the spatial–social interface has remained relatively siloed in the past, both spatial and social disciplines have rich intellectual histories with intertwined causes and consequences. We are excited to present this expansion of topics at the spatial–social interface, pushing the boundaries of theory, empirical knowledge, scale, methodology and integration. We believe that this collection serves to demonstrate the potential power and intrigue of the spatial–social interface as a topic, its central role in animal ecology, and the value in treating it as a unified field of research in its own right.

## Data Availability

This article has no additional data.
